# The Safety and Effectiveness of mRNA Vaccines Against SARS-CoV-2

**DOI:** 10.7759/cureus.45602

**Published:** 2023-09-20

**Authors:** Yahya F Jamous, Dalal A Alhomoud

**Affiliations:** 1 National Center of Vaccine and Bioprocessing, King Abdulaziz City for Science and Technology, Riyadh, SAU

**Keywords:** safety, efficacy, vaccines, mrna, sars-cov-2

## Abstract

The coronavirus disease 2019 (COVID-19) pandemic, caused by the novel severe acute respiratory syndrome coronavirus 2 (SARS-CoV-2), has resulted in numerous deaths worldwide, along with devastating economic disruptions, and has posed unprecedented challenges to healthcare systems around the world. In the wake of COVID-19's emergence in 2019, a variety of vaccine technologies were formulated and developed, including those that drew from the technology employed in messenger RNA (mRNA) vaccines, designed to curb the disease's transmission and manage the pandemic. mRNA vaccine has several advantages over traditional ones, and hence its development has received considerable attention recently.

Researchers believe the mRNA vaccine technology will emerge as the leading technology because it is potent, inexpensive, rapidly developed, and safe. This article provides an overview of mRNA vaccines with a special focus on the efficacy and safety of the Moderna and Pfizer-BioNTech mRNA vaccines against the different variants of COVID-19 and compare them with the Oxford-AstraZeneca (viral vector) and Sinopharm (inactivated virus) vaccines.

The clinical data reviewed in this article demonstrate that the currently authorized Moderna and Pfizer-BioNTech mRNA vaccines are highly safe and potent against different variants of COVID-19, especially in comparison with Oxford-AstraZeneca (viral vector) and Sinopharm (inactivated virus) vaccines.

## Introduction and background

Coronavirus disease 2019 (COVID-19) is a viral respiratory disease linked to severe acute respiratory syndrome coronavirus 2 (SARS-CoV-2). It was first detected in Wuhan in the Hubei Province, China, in late 2019, and rapidly spread across the world, prompting the World Health Organization (WHO) to declare COVID-19 a global pandemic in March 2020. COVID-19 has led to 5.94 million deaths until December 31, 2021, and brought with it devastating economic disruptions and unprecedented global health challenges [1,2]. As COVID-19 emerged, people and governments all over the world tried to combat the pandemic through isolation and quarantine measures to avoid spreading coronavirus while waiting for the vaccines against the condition to emerge.

The first phase I clinical trial of a messenger RNA (mRNA) vaccine, which encodes the spike (S) protein of SARS-CoV-2, took place in the United States (US) [3]. Between 1990 to 2010, there was a heady race in the medical community to develop a better delivery system for mRNA vaccines. The mRNA vaccine was initially tested in the early 1990s but not employed back then owing to several concerns, including those pertaining to its stability and immunogenicity. A study [4] demonstrated that mRNA stability can be controlled through optimization and formulation. In 1995, Dr. Pieter Cullis and his colleagues began studying lipid nanoparticles (LNPs) as a medium to deliver mRNA vaccines [5]. A study published in 2005 developed a process of safe synthetic mRNA, especially for injection into host cells [6]. A Canadian scientist employed modified mRNA to reprogram the adult cells into embryonic stem cells, leading to Moderna's creation in 2010 [7]. In 2010, Dr. Cullis, in collaboration with Dr. Katalin Karikó, started working on nanoparticle-mediated delivery of mRNA vaccines [8]. Dr. Corbett’s team, in association with Moderna, developed a COVID-19 mRNA vaccine in 2020 [9]. Pfizer-BioNTech and Moderna developed an mRNA vaccine for emergency use that displayed 94% efficacy in phase-III clinical trials. mRNA-1273 vaccine (non-replicating) was the first variant to undergo clinical trials [10]. Afterward, BNT162b2, the first COVID-19 mRNA vaccine produced by Pfizer-BioNTech, received approval in Canada and UK [3].

Despite the enthusiasm generated by the mRNA vaccine, it took a long time to reach the market due to some technical challenges. The biggest challenges involved the uptake of mRNA by host cells and the quick degradation of mRNA inside cells. Advancements in nanotechnology revolutionized the use of mRNA vaccines in clinical trials as they overcame the issue of mRNA vaccine delivery into the host cells. mRNA vaccines offer many benefits, including design flexibility, rapid production in large quantities, and the ability to generate an innate and adaptive immune response. mRNA vaccines are generally safe due to their transient expression and non-integrative nature inside the host cells. The key concerns associated with mRNA vaccines are their fragile stability and the generation of poor immune response [11].

Certain side effects (local and mild systemic reactions) and adverse events, including mortality, have been reported in recipients of mRNA vaccines [3,11]. Redness, swelling, heat, and pain are the most common local side effects of mRNA vaccines [12]. Identifying which immune signaling pathways are most effective in humans, along with how various species of animals respond to inflammatory signals, may improve the safety and efficacy of mRNA vaccines. Producing mRNA vaccines is relatively easy and inexpensive. Therefore, their development has received considerable attention recently [13,14]. This article reviews the safety and effectiveness of mRNA vaccines against SARS-CoV-2. The data that support the findings of this study are openly available on PubMed and the websites of WHO, the Centers for Disease Control and Prevention (CDC), and the European Medicines Agency (EMA).

In this comprehensive review, an extensive analysis of scientific literature and research articles related to mRNA vaccines was conducted, with a focus on their development, safety, and efficacy. Relevant studies were gathered from various sources, including PubMed, scientific journals, government agencies, and reputable international organizations. The primary objective of this review was to provide a detailed and up-to-date overview of mRNA vaccines, particularly those developed for SARS-CoV-2, also known as the novel coronavirus, which is responsible for the COVID-19 pandemic.

The search strategy aimed to include studies published until the cutoff period of September 2021, to ensure that as much information available about the subject was included. A combination of keywords such as "mRNA vaccines," "COVID-19 vaccine development," "mRNA vaccine safety," and "mRNA vaccine efficacy" was used to identify relevant articles. Additionally, references cited within selected articles were reviewed to further broaden the scope of the review.

## Review

mRNA vaccines: structure and production

**Figure 1:**
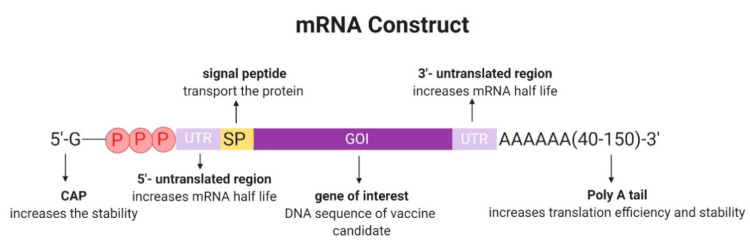
Typical mRNA construct* *[16]

The concept of mRNA vaccination involves the delivery of mRNA encoding viral proteins into immune cells to produce translated proteins. The translated proteins can be displayed on the surface of immune cells to be recognized by the immune system, which then starts making antibodies against the virus [15]. The final construct of mRNA vaccines contains an open reading frame (ORF), flanking untranslated regions (UTRs), a 5′ cap, and a poly(A) tail (Figure 1) [16].

There are two types of mRNA vaccines: self-amplifying mRNA (SAM) and non-replicating mRNA (NRM). They have a similar structure, including a 5′ cap sequence, 5′ and 3′ UTRs, an ORF carrying coding sequence, and a 3′poly(A) tail [17]. The main difference between them is in the presence of genetic replication machinery in SAM derived from flaviviruses or alphaviruses [18]. The synthesis starts by generating RNA from a plasmid DNA (pDNA) including an RNA polymerase promotor such as T7, and the sequence containing the mRNA construct. Plasmid DNA is linearized to transcribe the mRNA, which can then be degraded by incubation with DNase. The 5′ cap and the 3′ poly (A) tail can be added during in-vitro transcription or enzymatically after transcription [19]. Purification is a critical step after mRNA synthesis, which can be done using high-pressure liquid chromatography (HPLC), and the final product can be ready for sterility, identity, purity, and efficacy tests. These steps enable Good Manufacturing Practice (GMP) facilities to produce a new vaccine in a short period of time [19]. SAM is an advanced mRNA technology and has several advantages over non-replicating mRNA (NRM): (i) quick development, (ii) high levels of antigen expression, and (iii) enhanced T-cell-mediated immunity. 

Advantages of mRNA vaccines

mRNA vaccines have several advantages over traditional ones. First, they are potent due to their ability to regulate the adaptive immune system and induce both cellular and humoral immune responses [20]. They can also be self-adjuvant when combined with protamine called RNA active vaccine platforms that elicit a robust inherent innate immune response, a property that protein and peptide vaccines lack [21]. Modification of this kind of vaccine can improve its efficacy and stability [22], and the production procedure is completely cell-free, simple, and fast compared to the production of live attenuated and subunit vaccines [23]. This platform is also ideal for rapid responses to newly emerging pathogens because of its versatility and amenability to multiple targets [24]. In terms of safety, mRNA vaccines have the ability to avoid the potential risk of infection or insertional mutagenesis. mRNA vaccines can be degraded by normal cellular processes, and their in vivo half-life can be modulated by various modifications as well as through delivery techniques. To further improve the safety profile, the immunogenicity of the mRNA can be downregulated [20].

Delivery vehicles for mRNA vaccines

It is important to consider the mRNA vaccine delivery system because naked mRNA is not only susceptible to the breakdown of nuclei but is also too large and has a negative charge to pass through the cell membrane. Many important delivery vehicles are currently being investigated; all of them ensure the transport of mRNAs into the cytoplasm and protect them from being degraded by RNases. The delivery vehicle can have a synergistic adjuvant effect once added to some mRNA vaccines [24], and recently, tools derived from lipids and polymers have become the most widely used delivery carriers because they increase cellular uptake of RNAs [25]. The main delivery systems that could be used are the following:

Lipid-Based Delivery

LNPs are widely used as mRNA delivery carriers nowadays. Four main components are essential for constructing LNPs: (1) ionizable cationic lipid is important to control the virus-sized particles (~100nm) and ensure the transport of mRNA through the cytoplasm; (2) polyethylene glycol (PEG)-lipids are used to increase t½ of preparations; (3) cholesterol is useful to maintain the stability; and (4) the role of natural phospholipids is to provide a lipid bilayer structure. There are two main reasons for the wide use of LNPs as an mRNA vaccine delivery carrier. Firstly, they ensure the protection of RNA molecules by encapsulation. Secondly, LNPs effectively transport the mRNA into the cell cytosol by the mechanism of endocytosis [25]. The following safety concerns are associated with lipid-based delivery systems: (i) PEG-mediated delivery systems may elicit hypersensitivity reactions by activating the host complement systems; (ii) cytotoxicity of LNPs; (iii) triggering the production of reactive oxygen species and pro-inflammatory molecules. 

Polymer and Peptide-Based Delivery

The main goal of polymers is to protect RNA from degradation by RNase and to ensure intracellular delivery. However, during the formulation of polymer-based mRNA tools, many problems can arise due to their tendency to be highly polydispersable [26]. Structural modifications of polymer materials can be performed, such as the inclusion of lipid chains and hyperbranched groups to maintain the stability of the formulation and the safety of the profile [26]. Protamine is a natural cationic peptide that can be used for the encapsulation of the mRNA vaccine [27]. The addition of protamine to mRNA vaccines can improve their stability as well as their activity [28]. The following safety concerns are associated with polymer and peptide-based delivery systems: (i) high polydispersity, (ii) clearance of large molecules, and (iii) cytotoxicity due to cell stress. 

Considerations in mRNA vaccines

Stability and translation of mRNA are the two main factors in developing a successful RNA vaccine. Nowadays, the majority of vaccines are transported and stored in a continuous cold chain process, a condition that cannot be met in developing nations. Therefore, the development of mRNA vaccine stability is important [23]. Purity is also a key consideration as during translation the purity of the mRNA is essential. Any contamination with dsRNAs can lead to translation being inhibited and result in mRNA degradation. Many purification techniques such as HPLC and fast protein liquid chromatography (FPLC) could solve this problem [23].

SARS-CoV-2 and its variants

In general, coronaviruses are positive-sense single-stranded RNA viruses. SARS-CoV-2 is composed of the following four structural proteins: spike (S), envelope (E), membrane (M), and nucleocapsid (N) [29]. The spike (S) glycoprotein in SARS-CoV-2 is essential for cell entry and viral transmission. It is composed of two functional subunits, S1 and S2, which are subject to polybasic (furin) cleavage [26]. While S1 facilitates the host cell angiotensin-converting enzyme 2 (ACE2) surface receptor interaction, S2 is involved in membrane fusion [30]. The receptor-binding domain (RBD) is located on the S1 subunit at the S protein (amino acids 319-541); mutations in this region are critical and might induce phenotypical changes [31]. As expected, during the pandemic, multiple SARS-CoV-2 variants have been reported, such as Alpha, Beta, Gamma, Delta, and more recently, the Omicron variant. The evolution of SARS-CoV-2 within its emerging variants might affect the virus pathogenicity, transmissibility, antigenicity, and viral load [11].

The efficacy and safety of mRNA vaccines in the prevention of coronavirus

In the face of the sudden emergence of a new coronavirus pandemic, it is necessary to mention that the fast production of mRNA vaccines played a crucial role in the development of coronavirus vaccines [32]. This kind of vaccination can involve self-replicating RNA or mRNA, which both make cells expressing the SARS-CoV-2 spike protein. This process allows the body to identify and fight the corresponding pathogen. RNA vaccines mainly use nucleoside-modified messenger RNA co-formulated into LNPs, which protect the RNA strands and improve their insertion into the cells [11]. A number of mRNA COVID‑19 vaccines, including the Pfizer-BioNTech and Moderna vaccines, have been developed to use RNA for the stimulation of immune response [33]. RNA vaccines were the first authorized vaccines for the prevention of COVID‑19 in the United Kingdom, the United States, and the European Union [34,35]. The trial of the Pfizer/BioNTech vaccine (BNT162b2) involving 43,548 adult volunteers showed that the vaccine had 95% efficacy. The Moderna vaccine (mRNA-1273) trial enlisted a total of 30,420 volunteers and showed 94.1% efficacy [35]. This review focuses mainly on the Moderna and Pfizer/ BioNTech mRNA vaccines in terms of efficacy and safety.

Safety

No safety concerns have been identified related to either the BNT162b2 or Moderna vaccines. Usually, mild to moderate side effects are observed with both mRNA vaccines and they disappear a few days after vaccination [36]. The most common side effects reported in more than one in 10 people are as follows: headache, arthralgia, myalgia, diarrhea, fatigue, chills, swelling at the injection site, and pyrexia. Nausea, vomiting, and redness at the injection site were observed in less than one in 10 people. Other uncommon side effects were observed in less than one in 100 people, such as lymphadenopathy, insomnia, pruritus at the injection site, pain in extremities, and allergic reactions (urticaria, rash, and angioedema). Acute peripheral facial paralysis was rare, observed in less than one in 1,000 people. Available data revealed that anaphylaxis was very rare with both mRNA vaccines. As for all vaccines, close supervision and, if required, appropriate medical treatment are required for the administration of mRNA vaccines. Fifty-two participants reported an overdose in a clinical trial due to an error in dilution. Thus, overdose does not cause a higher reactogenicity or any side effect [37,38]. Anaphylaxis can occur after any vaccination, and rare cases of anaphylaxis have been reported in the recipients of mRNA COVID-19 vaccines [39]. If this occurs, close supervision and appropriate medical treatment are immediately required to treat the reaction. If a person has a history of anaphylaxis or acute allergic reaction to any ingredient in an mRNA COVID-19 vaccine PEG, they should switch to another type of vaccine. In addition, if anaphylaxis occurs after the first dose of an mRNA vaccine, the second dose should be withheld [40].

Efficacy

(A) Children: Once vaccine safety in adults had been established, both Pfizer-BioNTech and Moderna began studies with children aged five years and older. The FDA eventually approved the use of the Pfizer-BioNTech vaccine for this group (Table 1) [41]. Moderna collaborated. with the National Institute of Allergy and Infectious Diseases while developing the vaccine, and both are working together on the research, along with the Biomedical Advanced Research and Development Authority [42].

**Table 1:** Authorized vaccines by age groups* *[42]

Age, years	Pfizer	Moderna
≤4	No	No
5-11	Yes	No
12-17	Yes	No
≥18	Yes	No

Moderna began a study (KidCOVE) that involved children aged six months to under 12 years old to test the vaccine. It consisted of two parts, enrolling around 6,750 pediatric participants in the US and Canada. Each child was administered two doses of the Moderna vaccine with a gap of 28 days (GAVI, n.d.). In part 1, two doses were administered to children aged between two and 12 years (50 or 100 micrograms), while children under two years of age received two shots (25, 50, or 100 micrograms). Each group started with the lowest doses for the younger children to observe the reactions before giving the higher doses to older participants. Researchers then analyzed the results to determine which dose was safe and effective for each age group (GAVI, n.d.). In the second part of the study, the doses selected based on the above analysis or placebo shots (salt water) were administered to children. 

(B) Immunocompromised people: People with immunocompromising conditions or those on immunosuppressive medications or therapies could be at higher risk for severe COVID-19. Therefore, many medical organizations suggest that the COVID-19 vaccine could be given for these groups, such as the American College of Rheumatology (ACR) and the American College of Allergy, Asthma and Immunology (ACAAI). It is normal to exclude unhealthy people from trials because researchers need to first understand how the vaccines work in a healthy population (GAVI, n.d.). Therefore, data including the safety and efficacy of the vaccine in these groups are still limited. However, the approved COVID-19 vaccines are not live vaccines and can be safely used in immunocompromised people.

However, data remain scarce regarding people with HIV infection due to their exclusion from clinical trials. According to the CDC vaccine fact sheet, mRNA vaccines (Pfizer and Moderna) tend to be less effective in people who take medication that affects immune system function [43]. A study conducted in the US on transplant patients revealed that only 17% elicited an immune response after three weeks of being vaccinated with the Pfizer or Moderna vaccine. However, any available vaccine may still help in the prevention of severe diseases that may require hospitalization [43]. Data are still limited in terms of determining the ideal time for people who are planning to be on immunosuppressive treatment to be vaccinated. However, the information available so far suggests that receiving the vaccine two to four weeks before any administration of immunosuppressive therapies is safe. In case the patient is already on treatment, the American Society of Hematology advises waiting six months after therapy has been completed to increase the likelihood of developing immunity [44,45]; it has been shown that mRNA BNT162b2 vaccine is safe for the use of immunocompromised individuals. Another study has shown that the mRNA-1273 vaccine had high efficacy in immunocompromised patients with SARS-CoV-2 infections [46]. According to several studies, immunocompromised individuals respond poorly to mRNA vaccines and require a booster dose [47,48]. A recent study reported a low serologic response even after two doses of mRNA-1273 vaccine in immunocompromised patients with SARS-CoV-2 infections [48]. Numerous studies have compared the two mRNA vaccines (mRNA-1273 and BNT162b2) and found that the immune response generated by mRNA-1273 vaccine was lower than that generated by BNT162b2 vaccine in immunocompromised patients with SARS-CoV-2 infection [49,50].

(C) Pregnant or breastfeeding women: Pregnant women can become seriously ill while infected with COVID-19. Moreover, they are at higher risk of having preterm labor and many pregnancy complications, such as preeclampsia and coagulopathy. In fact, COVID-19 vaccination could protect pregnant women from becoming severely ill [51]. Pregnant or lactating women can receive any authorized vaccines especially as the Advisory Committee on Immunization Practices (ACIP) does not mention a particular vaccine. However, the Janssen vaccine should be avoided in pregnant, lactating, or postpartum women under 50 years of age to eliminate the risk of developing blood clots; instead, they can choose any available authorized vaccine such as the mRNA vaccine [52]. Data on the vaccine’s safety in pregnant women are limited as researchers exclude pregnant women from their trials. Only a very low percentage of participants became pregnant during the studies, and hence further investigations are ongoing. CDC has revealed that 30,000 women were pregnant during vaccination and detailed information such as symptoms that developed after vaccination were given by 1,800 pregnant women. Results showed that pregnant women experience the same side effects observed in non-pregnant women, and no miscarriages or any other complications have been reported [52]. Therefore, based on current evidence, COVID-19 vaccines appear to be safe in pregnant women with no teratogenic effects, and a pregnant woman cannot become infected if she has received any FDA-authorized COVID-19 vaccines. However, more data should be collected to assess the risks of COVID-19 vaccines in pregnant women and fetuses [51].

The vaccine can be given during any trimester of pregnancy. In addition, women should not postpone their pregnancy if they are planning to be vaccinated. Collected data suggests that authorized COVID-19 vaccines do not affect fertility, but some women have decided to receive the vaccine after the first trimester of pregnancy in order to avoid adverse reactions [53]. The safety of COVID-19 vaccines in breastfeeding women is still unknown, and data on their effect on breastfed infants are still limited. However, breastfeeding women can be given any available approved COVID-19 vaccines [51].

(D) People with allergies: Vaccines are considered to be the most effective tools to stop the spread of infectious diseases and decrease mortality [54]. However, as with any other drug, the development of an allergic reaction is possible in people who receive vaccines. Most of the reactions that have been reported so far are acute, but in rare cases, anaphylactic reactions can occur and cause serious complications. The main reason behind these reactions is the presence of excipients [55]. The rate of anaphylaxis reported by the CDC was 4.5 cases per million given doses, which reveals that anaphylaxis was rare with COVID-19 vaccination [39,56]. However, a prospective cohort study involving more than 60,000 employees reported a high rate of anaphylaxis associated with mRNA COVID-19 vaccines (2.47 per 10,000 doses) [57]. CDC guidelines state that "people with a history of severe allergic reactions not related to vaccines or injectable medications such as food, pet, venom, environmental, or latex allergies, still can get the COVID-19 vaccines”. Additionally, people suffering from allergies to any oral medications or those having a family history of severe allergic reactions may also get vaccinated [58]. The mechanism of allergic reaction to mRNA COVID-19 vaccine is largely unclear. However, it is believed to be due to the presence of excipients [59,60], such as polyethylene glycol used in the Pfizer and Moderna vaccines [61].

mRNA vaccines and sex-based differences

Immunity and vaccination outcomes (safety and efficacy) associated with vaccination are influenced by gender differences. It is well documented that females develop a more robust immune response to vaccination in contrast to males [62,63]; it has been reported that females have four times more allergic reactions to vaccination than males. Regulatory data for Moderna (mRNA1273), Pfizer-BioNTech (BNT162), Janssen (Ad26. COV*S), and AstraZeneca (AZD1222) mRNA vaccines against COVID-19 have reported sex-based differences in terms of safety and efficacy [64]. Non-regulatory data for Moderna (mRNA1273), Pfizer-BioNTech (BNT162), and Sputnik V (GamCOVID-Vac) mRNA vaccines have reported more efficacy in females in contrast to males [64]. Depending on sex, both hormonal and genetic variables can influence the immunological response to mRNA vaccines. According to one study, estrogen enhances the immune response in females while testosterone in males weakens the immune response to mRNA vaccines [65]. A study showed that more immune-related genes are located on the X chromosome than on the Y chromosome [66]. Studies have shown that females have a more robust immune response to mRNA vaccines due to the presence of two X chromosomes [67,64].

mRNA vaccines and obesity

As a major contributing factor to COVID-19 infection and poor prognosis of SARS-CoV-2 infection, obesity has been implicated as a serious risk factor [68]. It is also well-documented that obesity is responsible for a decreased immune response to mRNA vaccination [69]. Several lines of evidence have demonstrated the effects of obesity on mRNA vaccination in patients with COVID-19 infections [70,69]. It has been found that obesity weakens the antibody immune response to Pfizer-BioNTech (BNT162) vaccination in patients with SARS-CoV-2 infections. Non-adverse effects of obesity on vaccination efficacy have been reported in COVID-19 patients receiving Janssen (Ad26. COV*S) and Pfizer-BioNTech (BNT162) vaccines [71,3]. Another study reported that obesity marginally reduced vaccination efficacy in COVID-19 patients receiving the Moderna (mRNA1273) vaccine [12]. A population-based cohort study conducted in England reported the linear association between COVID-19 hospitalization and obesity in vaccinated individuals [72]. Table 2 presents an elaborate comparison between mRNA vaccines and other vaccines in terms of efficacy and safety [73,74].

**Table 2:** Comparison between RNA vaccines and other current vaccines in terms of efficacy and safety against COVID-19

Vaccine	Listed dosage	Efficacy	Safety	Compatibility with pregnancy and breastfeeding	Side effects
Pfizer-BioNTech mRNA BNT162 vaccine) (Strategic Advisory Group of Experts, 2020)	Two basic shots separated by 21 days, then a third booster shot after 6 months for all age stages except children under 5 years of age	• 95-97% against COVID-19, alpha, beta, and delta variants • 85% against Omicron variant after third dose	High safety impact on • Pregnant women • Hypertension • Diabetes • Asthma • Pulmonary • Liver and kidney disease; not safe for people with a history of severe allergic reaction to any component of it	• Proven to provide high safety rates for pregnant women • Highly recommended for breastfeeding women. Cannot cause COVID-19 infection in anyone, including the mother or the baby	• Headache • Muscleaches • Jointaches • Chills • Pain at the injection site • Tiredness • Swelling at the injection site • Fever
Moderna mRNA1273 vaccine (Organization, 2021)	Two doses separated by 8 weeks, then a booster dose after 4-6 months for all age stages except children under 12 years of age	• 94-95% against COVID-19, beta, and delta variants • 70-75% against Omicron after the booster dose	Reflects high safety on different types of diseases such as hypertension, diabetes, liver or kidney diseases, and asthma, but not safe for people with a history of severe allergic reaction or people who developed a reaction after the first shot	• Proven to have a high safety profile for pregnant women • The vaccine's efficacy in nursing mothers is likely to be comparable to that in other adults with no side effects	• Headache • Aches and pains in the muscles • Vomiting and nausea • Tiredness • Joint aches • Chills • Fever
Oxford- AstraZeneca (vectored) virus vaccine) [European Medicines Agency (EMA), 2021]	Two doses separated by 8 to 12 weeks and booster dose only for severely immunocompromised persons. All age stages except people under 18 years of age	• 75-76% against COVID-19 • 74% for alpha and beta variants, and 60% for delta variant; not proven yet for Omicron	After being rigorously tested on tens of thousands of patients, the Oxford-AstraZeneca vaccine was licensed for use; not safe for allergic people and people who have various blood problems as it might cause blood clots, in addition to people with capillary leak syndrome	• Unlike mRNA, there is minimal information on the use of AstraZeneca's vaccine in pregnant or breastfeeding women • Vaccination in pregnant womens should be undertaken only when the advantages of vaccination exceed its risk	• Nausea • Tiredness • Headache • Joint aches • Muscle aches • Chills • Fever • Might cause blood clots in certain cases
Sinopharm (inactivated vaccination) (SAGE Working Group on COVID-19 vaccines, 2021)	Two doses separated by 4 weeks and a booster dose for those who are older than 60. Anyone with a fever of 38.5oC or higher should postpone until the fever has subsided	• 79% efficacy against COVID-19 • 52% against delta and beta variants; not yet determined against Omicron variant	Generally safe with diabetes, hypertension, and asthma; safety-related data are limited regarding persons above 60 years of age or those below 16 years. In some rare cases, the vaccination might cause acute disseminated encephalomyelitis, a neurological disorder involving inflammation of the brain and spinal cord	There is not enough data yet available about pregnancy or breastfeeding in terms of the vaccine's compatibility but effects on pregnant women are expected to be comparable to those observed in non-pregnant women of similar age	• Cramps In the muscles • Allergies • Diarrhea • Cough • Muscle ache • Lethargy and joint discomfort • Tiredness • Nausea and vomiting

## Conclusions

mRNA vaccines are currently the focus of attention as a key tool for protection against COVID-19. Despite certain drawbacks such as strong immunogenicity and the potential for triggering irrelevant immune responses, the authorized mRNA vaccines are considered highly effective and safe. This is primarily due to their transient expression and non-integrative characteristics. Even though it is still too early to predict whether mRNA vaccines could offer full protection against COVID-19 and prevent the virus from spreading in the community, their safety and efficacy make this kind of vaccine very promising in comparison with other COVID-19 vaccines. Furthermore, determining which immune signaling pathways are most effective in humans along with how various species of animals respond to inflammatory signals may improve the safety and efficacy of mRNA vaccines.
